# Supplementary material for: Two membrane-bound transcription factors regulate expression of various type-IV-pili surface structures in Sulfolobus acidocaldarius
Source: PeerJ. 2019 Feb 26;7:e6459. doi: 10.7717/peerj.6459 (PMC6396743; doi:10.7717/peerj.6459)
Supplement: Supplemental Information 1 [file peerj-07-6459-s001.pdf]

Supplemental Information to Bischof et al.,

Table S1 Strains used in this study

Strain	Genotype	Reference
S. acidocaldarius MW001	*S. acidocaldarius* DSM639 Δ*pyrE* (*Saci*_1597; Δ91–412 bp)	(Wagner et al., 2012)
S. acidocaldarius MW328	*S. acidocaldarius* MW001 Δ*arnR*	(Lassak et al., 2013)
S. acidocaldarius MW330	*S. acidocaldarius* MW001 Δ*arnR1*	(Lassak et al., 2013)
E. coli Top 10	F- mcrA Δ(mrr-hsdRMS-mcrBC) φ80lacZΔM15 ΔlacX74 nupG recA1 araD139 Δ(ara-leu)7697 galE15 galK16 rpsL(Str^R) endA1 λ^-	Invitrogen
*E.coli*OverExpress(tm)C43(DE3)	F – *ompT hsdSB (rB- mB-) gal dcm* (DE3)	Lucigen

Table S2 Plasmids used in this study and their creation.

Plasmidnumber	Characteristics	*Reference*
pINIT_cat	FX sequencing vector for subcloning, chloramphenicol resistance, *sacB* selection cassette,	(Geertsma & Dutzler, 2011)
pSVA2533	pUC57-ArnR	codon optimized by Genscript, this study
pSVA2534	pUC57-ArnR1	codon optimized by Genscript, this study
pSVA2541	PINIT_cat:*arnR*, codon optimized *arnR* was amplified from pSVA2533 using primers 6292 and 6293 and cloned in pINIT_cat using *Sap*I.	This study
pSVA2542	PINIT_cat with *arnR1*, codon optimized *arnR1* was amplified from pSVA2534 using primers 6292 and 6293 and cloned in pINIT_cat using *Sap*I.	This study
pBXNH3	FX cloning *E.coli* expression vector with araBAD promoter and N-terminal 10x His tag and 3C protease cleavage site, amp resistance	(Geertsma & Dutzler, 2011)
pSVA2538	pBXNH3 backbone, for expression of codon optimized *arnR1*, generated by sub-cloning from pSVA2542 using *Sap*I.	This study
pSVA2543	pBXNH3 backbone, for expression of codon optimized arnR, generated by sub-cloning from pSVA2541 using *Sap*I.	This study

Table S3 Primers used in this study
* generated using http://www.fxcloning.org/

Primer	Characteristics /Reference	Sequence 5´-3´
6292	FX cloning primer ArnR fw, *Sap*I site *	atatatGCTCTTCtAGTacTaaAagcctgtttgatgtgctgaag
6293	FX cloning primer ArnR rev, *Sap*I site *	tatataGCTCTTCaTGCacggctcaggatttgcgcgaacacg
6294	FX cloning primer ArnR1 fw, *Sap*I site *	cagtcaGCTCTTCtAGTagcagcatgaataagcgtgtttttgac
6295	FX cloning primer ArnR1 rev, *Sap*I site *	tatataGCTCTTCcTGCAatacgAttgaagatttccgcaaagat
7785	fw primer *pupsX* with, 5´ Alexa Fluor 647 label, for MST	TCTCCCATATTACTATTGCCTATCTCGTTAATAGTATTTTC
7778	rev primer p*upsX*, for MST	GAAAATACTATTAACGAGATAGGCAATAGTAATATGGGAGA
7776	fw primer p*flaB*[41], with 5´ Alexa Fluor 647 label, for MST	ACGTCGATCAGCAGTCGAAAAATTGTGATTTTTGGTCGAAG
6205	rev primer p*flaB*[41], for MST	CTTCGACCAAAAATCACAATTTTTCGACTGCTGATCGACGT
7787	fw primer box 1, with 5´ Alexa Fluor 647 label, for MST	ATTGTGATTTTTGGTCGAAG
6201	rev primer box 1, for MST	CTTCGACCAAAAATCACAAT
7789	fw primer box 2, with 5´ Alexa Fluor 647 label, for MST	AACGTCGATCAGCAGTCGAA
6201	rev primer box 2, for MST	TTTCGACTGCTGATCGACGTT
9308	fw primer p*saci_2122*, with 5´ Alexa Fluor 647 label, for MST	ATAATTATTACTCAGCGTTTATAACGTTTAACATGTTAAAA
9309	rev primer p*saci_2122*, for MST	TTTTAACATGTTAAACGTTATAAACGCTGAGTAATAATTAT
9311	fw primer p*aapF* with 5´ Alexa Fluor 647 label, for MST	GTCTAAAGCCATAAAGATGAGCTTATTTTCATAGATTTAAGT
9312	rev p*aapF*, for MST	ACTTAAATCTATGAAAATAAGCTCATCTTTATGGCTTTAGAC
2073	qRT-PCR *upsX* fw (van Wolferen et al., 2013)	AATTTAGCATAGACCCAGCTTAC
2074	qRT-PCR *upsX* rev (van Wolferen et al., 2013)	ATTTACTACTGCCTTCAGCATAC
3512	qRT-PCR *aapF* fw (Henche et al., 2012a)	CTCCTGACTACCAACTGACTATTTATC

3513	qRT-PCR *aapF* rev (Henche et al., 2012a)	GTTCACCAGTAGAATAGCTCTTTACAC-
4764	qRT-PCR *adh (Saci_1690)* fw	GTAGAAAGTATCATCGTATACTGAAGTAC GAGTGAA
4765	qRT-PCR adh *(Saci_1690*) rev	ACCACTTCTCCTGCAAACTC

Table S4 Nucleotide sequence of codon optimized *arnR* and *arnR1*

Gene	Sequence
arnR	ATGACCAAGAGCCTGTTTGATGTGCTGAAGGAACTGGATA<br>GCCTGGTGGATTTTAGCCGTGCGAAGCTGCAATGGGATATT<br>CTGATTATCCTGGCGACCAAAGGTCCGAGCAGCACCACCG<br>AGATCAGCCAGACCATTAACACCAGCCGTAAGAGCATCAT<br>TGACGCGATTCGTAAACTGGTGGACAAAGAACTGGTGACC<br>AAGGTTAAACACGACATCTACGGTCTGAGCGATAAGGGCA<br>AAGAGCTGTGGAACAAAATTGACAGCGTGCTGAACATCGA<br>AGTTATTAACGGTAACAACCACAAGGGCCAGAGCAAAGAC<br>GAGGATATCCTGGCGCTGGAAAACCTGAGCCAATACTTCT<br>ATCTGATTAACCTGAGCAAGATGATCACCATTAACCACGAC<br>GGTCTGAGCCTGGATAAAGCGGCGCGTGAGCTGGGCGTTA<br>GCCGTCAGACCCTGAAGTACTATCTGGAACTGTTCGAGGA<br>AAAGAAACTGTTTCGTGTGATCGGCAAGCGTACCCACTTCA<br>AGAAAAACATCTACAAGTGCGTTCTGATGAACGAGGGCAA<br>ACGTCTGCTGTTCCGTCTGCCGGAATTTACCAAGATGAAAA<br>ACAACCTGCCGCTGAAACTGCTGCTGCGTCTGACCAACAGC<br>TACACCCTGGAGATGGCGAACGTGAAGGTTATGGGTTTCAT<br>CCTGATTAGCCTGCCGCTGCTGATGTATTTTCGTGATCAAC<br>TGGGCCTGATCGAACTGCCGTGGCTGTATGCGGTTATCTTC<br>CTGGCGCTGCTGAGCGTGTTCGCGCAAATCCTGAGCCGTTA<br>A
arnR1	ATGAGCAGCATGAATAAGCGTGTTTTTGACATTCTGCGTGA<br>ACTGGACAGCCTGGTTGACTTTAGCCGTGCGAAACTGCAAT<br>GGGACATCCTGATCATTCTGGCGACCAAAGGTCCGAGCAG<br>CACCACCGAGATCAGCCAGACCATTAACACCAGCCGTAAG<br>AGCATCATTGACGCGATTCGTAAACTGGTGGACAAAGAAC<br>TGGTGACCAAGGTTAAAGGTGACATCTACGGCCTGAGCGA<br>GAAGGGCGAAAAACTGCTGGAGAGCTTCGATAGCATCATG<br>AGCATTAACGTTACCGACAAACCGGATAGCAGCATCGAAA<br>GCAACAGCATTAGCCTGACCAACATCGCGGAGTACTTTTAT<br>ATGCTGGAAATTCTGAAGATGGCGCTGCTGAACAAACAGA<br>TCACCATTGACAAGGCGAGCCACGAGCTGGGTATTAGCAA<br>GCAAACCCTGAAATACTATATCGAAACCTTCACCGAAAAC<br>AAGCTGCTGAAAGTGGTTAACCAGGAAAGCGTTCTGGGCA<br>AGAGCAAGAAAATCTACGTGCTGACCGATGAGAGCCGTAA<br>ACTGGTTAGCCGTCTGCCGGAACTGACCCGTCTGAAGCGTA<br>ACCTGCCGCTGAAGATTCTGCTGAAACTGACCGGTAGCTAC

	CGTTATGAGATCGCGCTGACCAAAGTGATGCTGTTTAACGT<br>TATCAGCATTCCGGTGCTGATGTATCTGAAGGACCAACTGG<br>GCATCCTGGAAGCGATTTGGCTGTATGTTATTATCCTGCTG<br>CCGCTGCTGAGCATCTTTGCGGAAATCTTCAACCGTATCTA<br>A
